# Bisphenol A and phthalate endocrine disruption of parental and social behaviors

**DOI:** 10.3389/fnins.2015.00057

**Published:** 2015-03-03

**Authors:** Cheryl S. Rosenfeld

**Affiliations:** Bond Life Sciences Center, Genetics Area Program, Biomedical Sciences, University of MissouriColumbia, MO, USA

**Keywords:** EDC, bisphenol A, phthalate, xenoestrogen, rodent models, brain development, epigenetics, neuropeptides

## Abstract

Perinatal exposure to endocrine disrupting chemicals (EDCs) can induce promiscuous neurobehavioral disturbances. Bisphenol A and phthalates are two widely prevalent and persistent EDCs reported to lead to such effects. Parental and social behaviors are especially vulnerable to endocrine disruption, as these traits are programmed by the organizational-activational effects of testosterone and estrogen. Exposure to BPA and other EDCs disrupts normal maternal care provided by rodents and non-human primates, such as nursing, time she spends hunched over and in the nest, and grooming her pups. Paternal care may also be affected by BPA. No long-term study has linked perinatal exposure to BPA or other EDC and later parental behavioral deficits in humans. The fact that the same brain regions and neural hormone substrates govern parental behaviors in animal models and humans suggests that this suite of behaviors may also be vulnerable in the latter. Social behaviors, such as communication, mate choice, pair bonding, social inquisitiveness and recognition, play behavior, social grooming, copulation, and aggression, are compromised in animal models exposed to BPA, phthalates, and other EDCs. Early contact to these chemicals is also correlated with maladaptive social behaviors in children. These behavioral disturbances may originate by altering the fetal or adult gonadal production of testosterone or estrogen, expression of ESR1, ESR2, and AR in the brain regions governing these behaviors, neuropeptide/protein hormone (oxytocin, vasopressin, and prolactin) and their cognate neural receptors, and/or through epimutations. Robust evidence exists for all of these EDC-induced changes. Concern also exists for transgenerational persistence of such neurobehavioral disruptions. In sum, evidence for social and parental deficits induced by BPA, phthalates, and related chemicals is strongly mounting, and such effects may ultimately compromise the overall social fitness of populations to come.

## Introduction

The perinatal environment can dramatically shape later adult behaviors, and these disruptions can be propagated through transgenerational transmission to future generations (Rosenfeld, 2014). The impact of varying parental investment on offspring brain development is also gaining interest (Dulac et al., 2014; Rilling and Young, 2014). Disturbances in parental behaviors, such as nursing/feeding, huddling over, and grooming the neonates can have dramatic epigenetic and phenotypic consequences that persist for generations to come (Weaver et al., 2006).

Due to underlying neural disruptions, parental and social behaviors can be impacted by developmental exposure to endocrine disrupting chemicals (EDCs). Strong conservation exists in brain development and function across species spanning from rodents to humans (Rice and Barone, 2000; Howdeshell, 2002). In animal models and humans, neurobehavioral development is vulnerable to EDCs, because of the organizational-activational of endogenous steroidogenic hormones.

One of the most preeminent discoveries in neuroendocrinology in the last century was that sex steroid hormones, testosterone and estrogen, guide perinatal brain development in a sex dependent manner (Phoenix et al., 1959; Arnold and Breedlove, 1985; Morris et al., 2004). This early brain programming is termed the “organizational effects” of these steroid hormones. However, full elaboration of many sex-dependent behaviors requires a later surge of testosterone in adult males (“activational effects”). The organizational-activational effects of testosterone in many brain regions are due to aromatization to estrogen (Watson and Adkins-Regan, 1989; Konkle and McCarthy, 2011). Exposure to EDCs that disrupt these normal steroidogenic effects may thus result in neurobehavioral deficits, including social and parental behavioral deficits.

The two most common EDCs associated with parental and social behavioral disturbances are bisphenol A (BPA) and phthalates. Therefore, these two will be the primary focus, although, where appropriate, effects of other EDCs on these traits will be discussed. BPA is one of the most widely prevalent EDCs with production currently reported to be in excess of 15 billion pounds per year (Vandenberg et al., 2013; Grandviewresearch, 2014). BPA is present in a wide variety of commonly used products and applications, including polycarbonate plastics, the lining of metal food cans, certain dental sealants, thermal receipt paper, and many other items that are currently not required to be labeled that they contain BPA. The abundance of BPA has ensured extensive and longstanding exposure of animals and humans, including pregnant women (Environment Canada, 2008; Vandenberg et al., 2013). There is strong evidence that BPA is a neuroendocrine disruptor (Leon-Olea et al., 2014).

Di(2-ethylhexyl) phthalate (DEHP) is another ubiquitous EDC present in plastic products personal care products, paints, pharmaceuticals, food products, and textiles (Latini et al., 2003). Similar to BPA, this chemical can leach out of plastic products when heated. Its primary metabolite, mono-ethylhexyl-phthalate (MEHP) can also result in neuroendocrine disruption (Leon-Olea et al., 2014).

This review will first consider the evidence that BPA, phthalates, and other EDCs can alter parental care provided by the mother and the scant evidence to date by the father. Next, we will consider the impact of developmental exposure to these chemicals on social behaviors in animal models and linkages in humans. Finally, the potential mechanisms by which EDCs may affect these behavioral patterns will be explored.

## Evidence of endocrine disruption of parental behaviors

There have been no long-term studies examining for potential linkage of early exposure to these chemicals and later parental behavior deficits in humans. Therefore, this section will focus on the existing evidence from rodent and other animal models (Summarized in Table 1). A handful of rodent studies have reported that early exposure to EDCs can result in later effects on maternal care. General maternal behaviors assessed in these collective studies include the amount of time the female spends nursing, hunched over and in the nest, grooming, and latency to retrieve her pups.

**Table 1:** **Animal model and human epidemiological studies linking bisphenol A (BPA) and phthalates exposure to parental and social behavioral changes**.

**Publication(s)**	**Animal model/human cohort population**	**EDC(s) tested or correlation analysis performed**	**Dosing regimen/method to measure BPA concentrations**	**Major findings**
**PARENTAL BEHAVIORS**				
Palanza et al., 2002	CD1 female mice	BPA	Prenatal exposure of F_1_ offspring to 10 μg BPA/kg body weight (bw)/day or oil by oral administration through the F_0_ dam from days 14 to 18 of gestation. Some F_1_ adults (2–2.5 months of age) were also treated with 10 μg BPA/kg bw/day or oil and treatments spanned from days 14 to 18 of gestation. There were thus those F_1_ offspring who only received prenatal or adult exposure to BPA and those that were exposed to this chemical during the prenatal and adult periods resulting in four different treatment groups.	• F_1_ females exposed to BPA during the prenatal or adult period spent spend less time nursing and huddling over the pups but greater time engaged in nest building than controls and those that received BPA during both time periods.
Della Seta et al., 2005	Sprague-Dawley female rats	BPA	Adult F_0_ exposure to 40 μg BPA/kg bw/day by oral administration from the day after mating (gestation) through lactation (42 day duration).	Adult BPA exposure of F_0_ dams disrupts passive and active maternal care. These F_0_ females spend decreased time licking and grooming pups and a trend to less ano-genital licking. These F_0_ dams had reduced duration of time assuming an arched-back posture, which allows pups to suckle.
Boudalia et al., 2014	Wistar female rats	BPA	F_0_ adult exposure to 5 μg BPA/kg bw/day orally from the first day of gestation (GD1) through lactation (Post-natal day, PND 21).	F_0_ adult BPA exposed dams did not demonstrate any differences in incidence of resting inside or outside of the nest. Lifelong BPA exposure of F_1_ dams though resulted in them spending considerably less time outside of the nest. F_0_ dams did not demonstrate any differences in nursing position • F_1_ dams spend less time assuming “arched” and “blanket” nursing positions.
			F_1_ offspring then treated every two days with same dosing solution as received by the F_0_ mother from weaning (PND 21) until mating at adulthood (PND 100).	
Kundakovic et al., 2013	BALB/c mice	BPA	Adult exposure of F_0_ dams to one of the three oral doses of BPA (2, 20, 200 μg/kg bw/day) from gestational days 0 to 19.	Adult F_0_ females exposed to the highest dose of BPA spent more time licking, grooming, and nursing their pups in an arched back position. No effects were reported with the lower two doses.
Nakagami et al., 2009	Cynomolgus monkeys (*Macaca fascicularis*)	BPA	Male F_1_ infants exposed from gestational day 20 through term to 10 μg BPA/kg bw/day via osmotic pump.	BPA-exposed F_1_ male infant behavioral patterns in initiating maternal care became more reminiscent of female infants and subsequently these males were nursed less than control males. F_1_ females were not examined in this study.
**SOCIAL BEHAVIORS: ANIMAL MODELS**				
Williams et al., 2013	California mice (*Peromyscus californicus*)	BPA	Two weeks prior to breeding of the F_0_ dam through weaning at PND 30, they were exposed to 50 mg BPA/kg feed weight in the diet.	Perinatally BPA exposed F_1_ males show reduce territorial marking behavior when a control male was present in the testing arena. Perinatally BPA exposed F_1_ females showed decrease exploratory and increased anxiety-like behaviors.
			F_1_ males and females were placed on a control diet at weaning through adulthood.	
Ward and Blum, 2012	Native blacktail shiner fish (*Cyprinella venusta*) and introduced red shiner fish (*C. venusta*)	BPA	Adult F_0_ exposure for 14 days to BPA 1280 μg/L water (5.6 μM).	Adult BPA exposure of F_0_ fish disrupted normal visual communication signals and abolished species-dependent sexually selected behavioral traits. Hybridization occured between these two otherwise behaviorally isolated species.
Jasarevic et al., 2011	Deer mice (*P. maniculatus bairdii*)	BPA	Two weeks prior to breeding of the F_0_ dam through weaning at PND 21, they were exposed to 50 mg BPA/kg feed weight in the diet.	F_1_ control and perinatally BPA-exposed female selectively rejected F_1_ males prenatally exposed to BPA.
			F_1_ males and females were placed on a control diet at weaning through adulthood.	
Razzoli et al., 2005	Pair-bonded monogamous Mongolian gerbils *(Meriones unguiculatus)*	BPA and EE2	Adult F_0_ females treated daily with oral administration of BPA (2 or 20 μg/kg bw/day) or EE2 (0.04 μg/kg bw/day) from day of pairing to day 21 of cohabituation.	• BPA and EE2 adult exposed F_0_ females showed increase investigation of their F_0_ control male partner and reduced exploration.
Wolstenholme et al., 2013	C57Bl/6 mice	BPA	Prenatal exposure (7–10 days prior to F_0_ female being paired with a breeder male and for a 2 week duration) and ancestral exposure to 5000 μg/kg fw through the F_0_ maternal diet.	Prenatal and transgenerational exposure to BPA increased the amount of time juvenile F_1_and F_3_ male and female mice spent investigating a novel animal. F_3_ females ancestrally exposed to BPA persisted in investigating novel females, suggestive of impaired dishabituation.
Porrini et al., 2005	Sprague-Dawley rats	BPA	Daily oral administration of 4000 μg BPA/kg bw/day to adult F_0_ dams from gestation through lactation (Mating through weaning of pups at PND 21). F_1_ females were not exposed to BPA after weaning.	Perinatal exposure of F_1_ females exhibited reduced amount of time playing with males and engaging in social grooming. Findings are suggestive that developmental exposure to BPA may defeminize select social behaviors. F_1_ BPA exposed males were not assessed in this study.
Dessi-Fulgheri et al., 2002	Sprague-Dawley rats	BPA	Perinatal exposure of F_1_ females to BPA either at 40 mg/kg bw/day via daily maternal (F_0_) oral dosing from conception to weaning or 400 mg/kg bw/day from gestational day 14 to postnatal day 6.	Perinatally BPA-exposed F_1_ females, especially those exposed to the low dose, demonstrated a masculinized response in play behavior, as evident by their preference in playing with and engaging in socio-sexual exploration of other females. Perinatally BPA-exposed F_1_ males showed increase play behavior with females.
Wolstenholme et al., 2011b	C57Bl6J mice	BPA	Gestational exposure of F_1_ offspring to 1250 μg BPA/kg fw in the maternal diet.	• Gestational exposure of both F_1_ sexes resulted in increased play solicitations and approaches.
Jones et al., 2011	Long-Evans rats	BPA	Gestational (gestational day 7 to PND 14) and lactational exposure of F_1_ offspring to 50 μg/kg bw/day through daily maternal oral administration.	Perinatally BPA-exposed F_1_ males exhibited sexual behavioral deficits as adults. No disruptions were evident in the sexual behavioral of perinatally BPA exposed F_1_ females.
Monje et al., 2009	Inbred Wistar-derived rats	BPA	Neonatal exposure (PND 1–7) of F_1_ pups to 50 μg/kg bw/day or 20,000 μg/kg bw/day via daily subcutaneous injections.	Neonatal BPA exposed F_1_ females had later reductions in proceptive behaviors. Behaviors of neonatal BPA exposed F_1_ males were not assessed.
Panzica et al., 2005	Male Japanese quail (*Coturnix japonica*)	BPA	50, 100, and 200 μg BPA per egg.	*In ovo* BPA exposure did not affect F_1_ male copulatory behaviors. *In ovo* EB, DES, and genistein abolished copulatory behaviors of pubertal F_1_ males. F_1_ females were not assessed in this study.
		Estradiol benzoate (EB)	10 or 25 μg EB per egg.	
		DES	700 ng DES per egg.	
		Genistein	1000 μg genistein per egg.	
Wibe et al., 2002; Kaplan et al., 2013	Threespine stickleback (*Gastrosteus aculeatus*) and Mummichog (*Fundulus heteroclitus*)	Benzyl Butyl Phthalate (BBP)	Adult F_0_ exposure to 100 μg/L in the water (0.32 μM) daily for 26 days (threespine stickleback) or 4 weeks (mummichog).	BPP-treated threespine stickleback fish aggregated in a single shoal that remained at the bottom of the aquarium. Mummichog exposed fish preferred to shoal with smaller size fish. Potential sex differences were not assessed in this study.
Betz et al., 2013	Sprague-Dawley male rats	BPP	Five to six week old F_0_ males were provided 5000 and 10,000 mg/L in the drinking water (0.16 and 0.032 μM) until they were 20–21 weeks of age (15 week duration of exposure).	Juvenile to adult BPP exposed F_0_ males displayed aberrant social behaviors. F_0_ females were not assessed in this study.
Lee et al., 2006	Wistar-Imamichi rats	Di-n-butyl phthalate (DBP)	Perinatal exposure of F_1_ offspring through the maternal diet from gestational day 15 to weaning (PND 21) to: DBP- 20, 200, 2000, and 10,000 mg/kg bw/day DINP- 40, 400, 4000, and 20,000 mg/kg bw/day DEHA- 480, 2400, and 12,000 mg/kg bw/day	DBP, DINP, and DEHA at several doses reduced copulatory behavior in perinatally exposed F_1_ males. All doses of DBP, DINP, and DEHA decreased the lordosis quotient in perinatally exposed F_1_ females.
		Diisononyl phthalate (DINP)		
		Di-2-ethylhexyl adipate (DEHA)		
**SOCIAL BEHAVIORS: HUMAN EPIDEMIOLOGICAL STUDIES**				
Perera et al., 2012	87 boys and 111 girls spanning 3–5 years of age	Maternal urinary BPA concentrations	Median maternal urinary concentrations at 34 weeks gestation: 1.8 μg/L (7.9 nM) Median child urinary concentrations at 3–4 years of age: 3.5 μg/L (15.3 nM)	Linkage of prenatal BPA concentrations and increased emotionally reactive and aggressive behaviors in boys. Opposite effect in girls with increased exposure to BPA correlating with decreased anxiety and aggressive behaviors.
Evans et al., 2014	77 boys and 76 girls spanning 6–10 years of age	Maternal urinary BPA concentrations	Median maternal urinary BPA from 10 to 39 weeks gestation with mean gestational age at collection = 26.6 weeks: 1.1 μg/L (4.8 nM)	• Increased maternal urinary BPA concentrations linked with greater externalizing and aggressive behaviors in boys.
Braun et al., 2009	249 mothers and their 2-year old children	Maternal urinary BPA concentrations	Median maternal urinary concentrations: 1.3–1.8 μg/L (5.6–7.9 nM), as measured at 16 and 26 weeks gestation and at birth	• Linkage of prenatal BPA concentrations (as determined by maternal urinary concentrations during pregnancy) and externalizing behaviors for girls but not boys.
Miodovnik et al., 2011	404 mothers and their 7–9 years of age children	Maternal urinary BPA and 10 individual phthalate metabolite concentrations	Maternal urine was collected between 25 and 40 weeks gestation (mean = 31.2 weeks).	Linkage with phthalate exposure and later childhood social impairments, including social cognition, communication, and awareness. No associations observed between BPA exposure and these behavioral disturbances.
			Median BPA concentration: 1.3 μg/L (5.6 nM)	
			Median phthalate concentrations ranged from 1.6 to 430 ng/ml	
Lien et al., 2014	122 mothers and their 8 year old children	Maternal urinary phthalate concentrations (7 different forms)	Maternal urine was collected in the third trimester of gestation. Geometric mean range of urinary phthalate metabolite concentrations: 26.8–109.1 μg/g creatinine	• Positive correlation with perinatal exposure to phthalates and delinquent and aggressive behavioral scores.
Kobrosly et al., 2014	153 mothers and their 6–10 year old children	Maternal urinary phthalate concentrations (monoisobutyl phthalate, MiBP, and monobenzyl phthalate, MBzP)	Maternal urine was collected between 10 and 39 weeks gestation (mean = 26.6 weeks)	Boys of mothers with higher urinary monoisobutyl phthalate concentrations were more likely to show higher scores for inattention, rule-breaking behavior, aggression, and conduct problems. Increased concentration of monobenzyl phthalate was correlated with conduct problems in boys but reduced anxiety scores in girls.
			Median of MiBP = 1.0 ng/ml (4.5 pM)	
			Median of MBzP = 3.4 ng/ml (13.3 pM)	

CD-1 female mice exposed orally during the fetal and/or adult period to BPA (10 μg/kg body weight (bw)/day for gestational days 14–18) spend less time nursing their pups and huddling over them while in the nest (Palanza et al., 2002). Acute exposure of adult female Sprague-Dawley rats exposed to BPA (40 μg/kg bw, orally per day) from mating to weaning of their pups disrupts both active and passive maternal care, manifested by decreased time licking and grooming the pups, a trend to less ano-genital licking, and less time assuming an arched-back posture to allow the pups to suckle (Della Seta et al., 2005). Similarly, Wistar female rats treated with 5 μg BPA/kg bw/day from the first day of gestation through lactation (postnatal day, PND 21) decreased the amount of time they nursed and attended to their pups (Boudalia et al., 2014). Another study tested the effects of gestational exposure (0–19 days) to three oral doses of BPA (2, 20, and 200 μg/kg bw/day) on maternal care provided by BALB/c mice. In contrast to the prior studies, the highest dose of BPA tested increased the amount of time females spent licking and grooming and nursing their pups in an arched back position (Kundakovic et al., 2013).

These conflicting rodent model findings may be attributed to various factors. First, all four of these above studies used rats or mice of different strains, and there is evidence of strain-specificity and phenotypic differences in response to BPA and other estrogenic EDC (Spearow et al., 1999, 2001; Kendziorski et al., 2012). The administered dose and timing, duration of exposure (perinatal vs. adult), and corresponding generational differences (F_0_ vs. F_1_) may also be potential factors. Finally, differences in animal husbandry (composition of the cages and water bottles, diet-phytoestrogen or non-phytoestrogen free, and shavings-corn cob or aspen) might account for the disparate rodent results.

Infants may influence, through vocalizations, direct contact, and other forms of communication, the amount of parental care provided to them with males tending to initiate and receive more parental investment (Della Seta et al., 2005; Hao et al., 2011). Early EDC exposure though may alter an infant's ability to stimulate maternal care provided to them. For instance, the behavioral patterns of Cynomolgus monkeys (*Macaca fascicularis*) male infants prenatally (gestational day 20 through term) exposed to BPA (10 μg/kg bw/day via osmotic pump) were more reminiscent of females, and these mothers nursed their “feminized” sons less than those rearing control males (Nakagami et al., 2009).

While there are no studies to date on whether acute and developmental exposure to phthalates affects maternal care, other EDCs have been reported to disrupt these behavioral patterns. Long-Evans (LE) rat pups prenatally exposed through their biological dam to the environmental chemical 3, 4, 3′, 4′-tetrachlorobiphenyl (PCB 77, 2000 μg/kg bw/day on gestational days 6 through 18 via daily subcutaneous injections) resulted in their foster dams spending an increased frequency nursing them and allogrooming (Cummings et al., 2005). This cross-foster approach further revealed the complex interactions exist between maternal and fetal exposure to PCB 77 and amount of time foster dams spent in the nest and grooming the pups, along with decreased duration engaged in high-crouch nursing. Perinatal exposure (either 3 days after the dam was paired with a male or 3 days after parturition of a previous litter through weaning of pups) via daily maternal oral dosing to diethylstilbestrol (DES, 0.2 μg/kg bw/day) or methoxychlor (MXC, 2000 mg/kg bw/day) of monogamous female pine voles did not however affect their later maternal care behaviors (Engell et al., 2006).

The impact of EDCs on later paternal care provided is largely unknown, probably because few mammalian species exhibit biparental care (Clutton-Brock, 1989). Yet, disturbances in paternal care may have dramatic epigenetic and phenotypic consequences that persist for several generations (Bredy et al., 2007; Braun and Champagne, 2014; McGhee and Bell, 2014). Even so, there have been no published rodent studies to date on how early exposure to EDCs affects paternal care. However, in polygynous sand gobies (*Pomatoschistus minutus*), where the male is responsible for tending the eggs, endocrine disruption of paternal care behaviors has been reported (Saaristo et al., 2010). Polygynous sand goby males compete for females and then assume responsibility for nest building and attendance. However, adult males exposed for 1–4 weeks to 11 ng/L water (36.7 pM) of 17α-ethinyl estradiol (EE2, estrogen in birth control pills) showed suppressed nest building activity, along with decreased courtship behaviors. These combined deficiencies thus likely effect successful fry rearing by exposed males. Future work is needed in monogamous and biparental rodent and other animal models to determine how developmental exposure to EDC affects paternal care and parenting providing by his partner.

## Evidence that endocrine disruptors affect social behaviors

Extensive data from retrospective human studies and animal models links perinatal exposure to EDCs and later social deficits (Table 1). The evidence in animal models will first be considered. Social behaviors to be considered include any that involve an interaction between difference members of the same species, such as various forms of communication, mate choice, pair bonding, social inquisitiveness and recognition, play behavior, social grooming, copulation, and aggression.

## Animal models

Animal communication assumes various forms besides direct vocalizations. In California mice (*Peromyscus californicus*), males use territorial marking to communicate and protect their home range and mate from intruders. Males pericoceptionally through perinatally exposed (2 weeks prior to breeding of the dam through weaning at PND 30) to BPA (50 mg/kg feed weight in the maternal diet) show reduction of this behavior when a control male is present in the testing arena (Williams et al., 2013). BPA exposure (1280 μg/L water, 5.6 μM for 14 days) of native blacktail shiner fish (*Cyprinella venusta*) and introduced red shiner fish (*C. venusta*) disrupts normal visual communication signals and abolishes the species-dependent sexually selected behavioral traits with the net potential for hybridization to occur between these two otherwise isolated species (Ward and Blum, 2012). Sprague-Dawley female rats prenatally exposed from gestational days 16 to 18 via daily intraperitoneal injection to the PCB mixture Aroclor (A) 1221 (100 μg/kg, 1000 μg/kg, and 10,000 μg/kg bw/day) have later vocalization deficits, as well as changes in mating patterns, movement and likelihood to mate (Steinberg et al., 2007).

Mate choice and pair-bonding formation are vulnerable to endocrine disruption. In mate choice trials, control and BPA-exposed female deer mice (*P. maniculatus bairdii*) selectively reject males developmentally exposed to BPA (50 mg/kg feed weight through the maternal diet) (Jasarevic et al., 2011). Likewise, female F_3_ Sprague-Dawley rats prefer F_3_ males whose ancestors were not prenatally exposed to the anti-androgenic EDC, vinclozolin (100 mg/kg bw/day from gestational days 8 to 14 via daily intraperitoneal injection) (Crews et al., 2007). Female sand gobies favor mating with control males, as opposed to those exposed for 7–24 days to EE2 (4 ng/L water, 13.3 nM) (Saaristo et al., 2009). Pair-bonded monogamous Mongolian gerbil (*Meriones unguiculatus*) females treated for a 3 week duration (from pairing to day 21 of cohabituation) with daily oral administration of either BPA (2 or 20 μg/kg bw/day) or EE2 (0.04 μg/kg bw/day) exhibit heightened investigation of their partner and less exploration than untreated controls (Razzoli et al., 2005).

Introduction of new animals into the habitat results in social inquisitiveness and in time recognition of the new member with reduced bouts investigating this individual, i.e. habituation. Developmental and transgenerational exposure to BPA (5000 μg/kg feed weight through the F_0_ maternal diet 7–10 days prior to being paired with a breeder male and for 2 weeks thereafter) prolonged the duration of time juvenile F_1_ and F_3_ C57BL/6 mice, respectively, spend investigating a novel animal (Wolstenholme et al., 2013). Further, F_3_ females, whose ancestors were exposed to BPA, persisted in investigating novel females longer than controls, suggestive of impaired dishabituation.

Exposure to BPA may affect other socio-sexual behaviors, including play, social investigation, copulation, and aggression. Decrease amount of play with males and social grooming were evident in female Sprague-Dawley rats perinatally exposed to BPA (4000 μg/kg bw/day to the dams via daily oral administration from mating through weaning at PND 21), which indicates that this chemical can defeminize select female social behaviors (Porrini et al., 2005). Sprague-Dawley rats exposed perinatally to BPA (40 mg/kg bw/day from conception to weaning or 400 mg/kg/day from gestational day 14 to postnatal day 6 via daily maternal oral dosing) demonstrated masculinization of female social behaviors with such females selectively preferring to play with and engage in socio-sexual exploration of other females (Dessi-Fulgheri et al., 2002). Another study though with C57Bl6J mice suggests that gestational exposure to 1250 μg BPA/kg feed weight increased play solicitations and approaches by both sexes (Wolstenholme et al., 2011b).

Sexual behaviors are also vulnerable to the early effects of BPA. Male LE rats, who received a “*low dose*” BPA (50 μg/kg bw/day through maternal oral administration spanning gestation and lactation (gestational day 7 through PND 14), were sexually incompetent as adults, but no deficits in female sexual behavior were noted in this study (Jones et al., 2011). In contrast, another report found that neonatal exposure of inbred Wistar-derived female rats to BPA (50 μg/kg bw/day or 20,000 μg/kg bw/day via daily subcutaneous injections to the pups) had later reductions in proceptive behaviors (Monje et al., 2009). While BPA exposure (50, 100, and 200 μg per egg) did overtly effect male Japanese quail (*Coturnix japonica*) copulatory behaviors, *in ovo* treatment with other estrogenic chemicals, including estradiol benzoate (10 or 25 μg per egg), DES (700 ng per egg), and the soy phytoestrogen, genistein (1000 μg per egg), abolished this behavior in pubertal males (Panzica et al., 2005).

Current data demonstrate that phthalates can also affect varying social behaviors in rodents and fish. Benzyl butyl phthalate (BBP) exposure through the water (100 μg/L, 0.32 μM) altered the shoaling behaviors (collection of fish for social purposes) in threespine stickleback (*Gastrosteus aculeatus*), who were treated each day for 26 days, and mummichog, a small killifish, (*Fundulus heteroclitus*), who were treated daily for 4 weeks (Wibe et al., 2002; Kaplan et al., 2013). BPP-treated threespine stickleback fish tended to aggregate in a single shoal that remained at the bottom of the test aquarium; whereas mummichog exposed fish preferred to shoal with smaller size fish. Male Sprague-Dawley rats exposed from adolescence to adulthood (5–6 weeks of age to 20–21 weeks of age) to BPP through the drinking water (5000 μg/L and 10, 000 μg/L or 0.016 and 0.032 μM, respectively) displayed aberrant social behaviors (Betz et al., 2013). Male and female Wistar-Imamichi rats developmentally exposed through the maternal diet from gestational day 15 through weaning (PND 21) to various phthalate chemicals (di-n-butyl phthalate, DBP- 20, 200, 2000, and 10,000 mg/kg bw/day; diisononyl phthalate, DINP- 40, 400, 4000, and 20,000 mg/kg bw/day; di-2-ethylhexyl adipate, DEHA- 480, 2400, and 12,000 mg/kg bw/day) exhibited sexual behavioral deficits (Lee et al., 2006). Copulatory behavior was diminished in male rats exposed to the varying doses of these chemicals. Similarly, the lordosis quotient was reduced in all treatment group females.

## Human epidemiological studies

Associations between early BPA exposure and social behavioral disruptions in children are generally based on urinary BPA concentrations in the mother or child (Table 1). An African-American and Dominican women and their children population cohort that included 87 boys and 111 girls spanning 3–5 years of age showed higher exposure of BPA correlated with increased aggressive behaviors in boys but decreased aggression in girls (Perera et al., 2012). A more recent study with 153 six to ten year old children (77 boys and 76 girls) also demonstrated similar sex differences in vulnerability with increased maternal urinary BPA concentrations linked with greater externalizing and aggressive behaviors in boys (Evans et al., 2014). However, a study examining 249 mothers and their 2-year old children reported elevated prenatal BPA exposure associated with greater externalizing (aggression and hyperactivity) in girls but not boys (Braun et al., 2009).

Perinatal exposure to phthalates is also correlated with social behavioral disturbances. A study population of 404 mothers and their 7–9 years of age children pairs linked early contact to this chemical and later childhood social impairments, including social cognition, communication, and awareness (Miodovnik et al., 2011). This study however did not find any association between BPA exposure and effects on the examined social behaviors or sex-dependent differences. Examination of 122 mother-8 year old child pairs from Taiwan showed positive correlation of delinquent and aggressive behavioral scores and early exposure to different phthalate chemicals, as evidenced by maternal urinary concentrations (Lien et al., 2014). However, this study did not examine for potential sex-dependent differences. Another study with 153 mothers and their 6–10 year old children indicates that prenatal phthalate exposure is associated with later sex-dependent behavioral disturbances (Kobrosly et al., 2014). Boys of mothers with higher urinary monoisobutyl phthalate concentrations were more likely to show higher scores for inattention, rule-breaking behavior, aggression, and conduct problems; whereas increased concentration of monobenzyl phthalate was correlated with conduct problems in boys but reduced anxiety scores in girls.

## Mechanisms by which endocrine disruptors may affect parental and social behaviors

### Disruption of steroid hormone production or at the steroid receptor level

**Figure 1:**
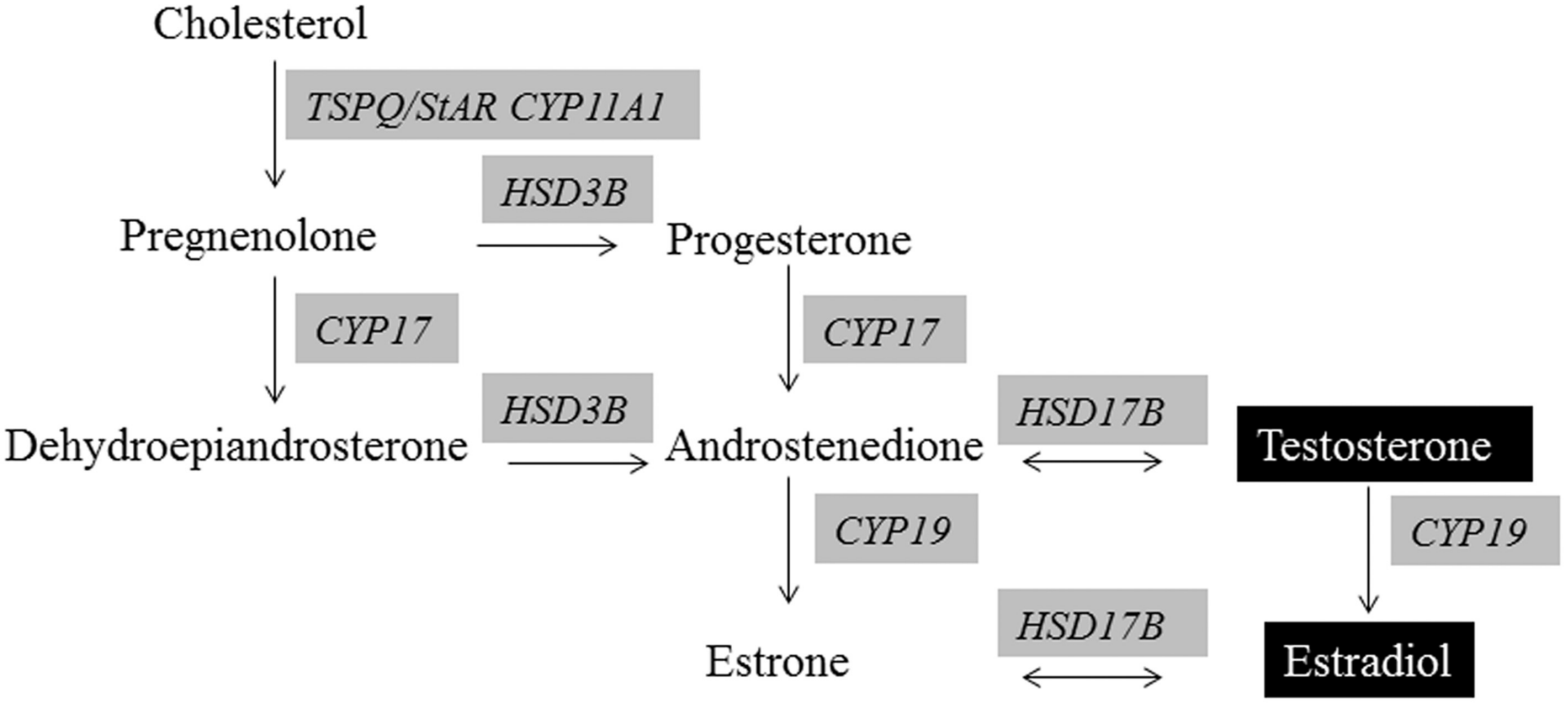
**Steroidogenesis of androgens and estrogens**. Evidence exists that all of the shaded enzymes required in the synthesis of these hormones are altered by BPA and/or phthalate exposure.

As detailed above, full elaboration of many social and parental behaviors is dependent on the organizational and activational effects of testosterone and estrogen (Phoenix et al., 1959; Arnold and Breedlove, 1985; Morris et al., 2004). Figure 1 illustrates the primary factors and enzymes required for normal testosterone and estrogen production. Both BPA and phthalates can affect steroidogenesis at several points in the pathway for males and females. Steroidogenic acute regulatory protein (StAR, STARD1) is an essential mitochondrial protein for transporting cholesterol into the cell and is thus considered the rate-limiting step for steroid hormone production. BPA suppresses *StAR* mRNA expression in male and female gonads of rodents and fish (D'Cruz et al., 2012; Horstman et al., 2012; Liu et al., 2012; Peretz and Flaws, 2013; Savchuk et al., 2013). Expression of gonadal steroidogenic enzymes, including *Cyp11*, *Hsd3b*, *Hsd17b*, *Cyp17*, *Cyp19* is also generally inhibited by BPA exposure (D'Cruz et al., 2012; Liu et al., 2012; Nanjappa et al., 2012; Peretz and Flaws, 2013; Savchuk et al., 2013).

Most animal model studies report BPA exposure decreases production of testosterone (T) and estrogen (E2) by male and female gonads (Akingbemi et al., 2004b; Peretz et al., 2011; D'Cruz et al., 2012; Nanjappa et al., 2012). However, a study with isolated porcine ovarian follicles suggests potential dose-dependent effects with lower dose BPA concentrations (0.1 mM) elevating E2; whereas large doses (1 and 10 mm) suppressing E2 levels (Grasselli et al., 2010). *In ovo* BPA treatment (0.01 or 1.4 ppm) of *Caiman latirostris* eggs elevated E2 and lowered T concentrations and concomitantly reversed the normal temperature sex determination mechanisms with a predominance of females occurring in these groups (Stoker et al., 2008). In girls with precocious puberty, elevated urinary BPA concentrations were associated with elevated T, E2, and pregnenolone levels (Lee et al., 2014). Phthalate exposure is also associated with decreased StAR and steroidogenic enzyme expression and steroid hormone production (T and E2) by the male gonad (Akingbemi et al., 2001, 2004a; Svechnikov et al., 2008; Botelho et al., 2009; Chauvigne et al., 2011; Desdoits-Lethimonier et al., 2012; Saillenfait et al., 2013; Beverly et al., 2014), as well as circulating concentrations of T and E2 in men (Meeker et al., 2009). In contrast, one report identifying disrupted social interactions in phthalate-treated rats indicated that this chemical increased E2 concentrations (Betz et al., 2013).

BPA and phthalates may also disrupt normal organizational/activational steroidogenic effects by altering the expression of the cognate receptors, ESRs and AR, in the neural regions governing social and parental behaviors, namely the hypothalamus. In this region, developmental exposure to BPA has been shown to alter the expression of ESR1 and ESR2 in rodent and ovine models, although the directionality is seemingly dependent upon sub-region, dose, time of assessment, and possibly species (Ramos et al., 2003; Ceccarelli et al., 2007; Monje et al., 2007; Mahoney and Padmanabhan, 2010; Cao et al., 2012, 2013, 2014; Patisaul et al., 2012; Kundakovic et al., 2013). Scant information exists on how phthalates may affect neural expression of ESRs and AR. The aforementioned rat phthalate study above also showed increased ESR1 expression in the amygdala of rats treated with 5 mg/L (0.16 μM) BBP (Betz et al., 2013). Combined treatment of 4 wk old rats with BPA (285.4 mg/kg) and DBP (285.4 mg/kg) in the feed increased AR expression in the brain (Zhang et al., 2013), which might render these animals more sensitive to endogenous and exogenous androgens.

### Disruption of the hypothalamic-pituitary-adrenal (HPA) axis

BPA, phthalates, and other EDC exposure may disturb parental and social behaviors through disruptions in the classic stress-associated adrenocortical axis. One rat study showed that early BPA exposure (oral administration of 40 μg/kg bw/day to the dam throughout gestation and lactation) led to sex dependent effects on circulating corticosterone concentrations (elevated in BPA-exposed females) and neural (hippocampal expression) of *Gr* (lower expression in BPA-exposed males) (Poimenova et al., 2010). Further, BPA-treated female rats developmentally exposed to this same dosing regimen exhibited increased basal corticosterone and suppressed hypothalamic *Gr* expression (Panagiotidou et al., 2014).

Another study in rats where dams were subcutaneously injected with 2 μg BPA/kg bw/day from gestational day 10 to lactational day 7 showed sex-dependent alterations in the adreno-cortical axis (Chen et al., 2014). BPA-exposed male rats had increased basal concentrations of serum corticosterone and adrenocorticotropin (ACTH) and corticotropin-releasing hormone (*Crh*) mRNA expression, but these changes were not evident in exposed females. Following subsequent exposure to a mild stressor, corticosterone and ACTH concentrations increased further in BPA males; whereas, these hormones decreased in BPA females. In non-stressed animals, *Gr* mRNA was increased in the hippocampus and hypothalamic paraventricular nucleus of BPA females. In contrast, these transcripts decreased in BPA males. The collective results suggest that BPA exposure might affect the HPA axis in males and females but the specific effects are sex-dependent.

Phthalates also appear to disrupt the HPA axis. Oral exposure to DEHP (500 mg/kg bw/day for 4 days) increased concentrations of ACTH and corticosterone in treated male rats at 20 and 40 days of age; whereas no effects were observed in adult (60 days of age) males (Supornsilchai et al., 2007). Isolated adrenocortical cells from the 20 and 40 day old treated rats were more sensitized to the effects of ACTH, dibutyrl cAMP, and 22R-hydroxylase, as evidenced by increased production of corticosterone and ACTH treatment stimulated greater transport of endogenous cholesterol into the mitochondria.

### Disruption of neuropeptide/protein hormones and their cognate receptors

In the mouse and rat, central oxytocin receptors (OXTR) are obligatory for the expression of maternal behavior, and variation in OXTR levels in the medial preoptic area of the hypothalamus (MPOA) are functionally linked to differences in degree of maternal care (Champagne et al., 2006; Weaver, 2007). California mice fathers are known to exhibit increased oxytocin immunoreactivity compared to non-paternal males (Lambert et al., 2011). Increased signaling through the OXTR is correlated with high degree of maternal behavior, such as pup licking and grooming, while ESR1 is an important regulator of OXTR expression (Champagne et al., 2006). Prolactin, oxytocin, and vasopressin have been implicated in paternal care in California mice, monogamous prairie voles (*Microtus ochrogaster*), and humans (Gubernick and Nelson, 1989; Parker and Lee, 2001; Bales et al., 2004; Wynne-Edwards and Timonin, 2007; Gordon et al., 2010). Social behaviors are also dependent upon oxytocin, vasopressin, and protein binding to their cognate receptors in the brain (Carlson et al., 2006; Benarroch, 2013; Walker and McGlone, 2013; Wudarczyk et al., 2013; Babb et al., 2014; Lieberwirth and Wang, 2014).

Brains from 18.5 days post-coitus (dpc) mice exposed to BPA express less oxytocin, OXTR, and vasopressin than control males (Wolstenholme et al., 2011a, 2012). Reductions in brain expression for vasopressin persists in transgenerationally BPA exposed F_4_ males and females, and oxytocin is decreased in F_4_ males (Wolstenholme et al., 2012). Another study, however, suggests that in the paraventricular nucleus (PVN), oxytocin-immunnoreactive neurons increase in neonatally exposed BPA female Long Evans rats (Adewale et al., 2011), but non-pregnant, non-lactating females were examined. A recent study with juvenile prairie voles showed neonatal exposure to BPA increased AVP-immunoreactive neurons in the anterior PVN but decreased OT-immunoreactive neurons in this same region (Sullivan et al., 2014). *In vivo* and *in vitro* studies suggest that BPA increases PRL levels in male and female rats (Steinmetz et al., 1997; Goloubkova et al., 2000; Ramos et al., 2003; Delclos et al., 2014). Likewise, phthalate exposure is associated with elevated prolactin production in rats of both sexes and men (Lee et al., 2004; Li et al., 2011). No previous study has assessed the effects of BPA or phthalate-exposure on PRL in parenting males and females.

### Epimutations

Epimutations are the most plausible mechanisms by which early exposure to BPA, phthalates, and other EDCs may lead to later neurobehavioral disturbances. Such changes may include alteration in DNA methylation, histone proteins, non-coding RNA, or chromatin arrangement. It is now apparent from a variety of animal model studies that BPA, phthlates, and other EDCs can lead to DNA methylation and corresponding gene expression changes in a variety of tissues, including the brain (Yaoi et al., 2008; Jang et al., 2012; Tang et al., 2012; Kundakovic et al., 2014; Zhao et al., 2014; Martinez-Arguelles and Papadopoulos, 2015).

Only two studies to date have shown BPA-induced DNA methylation changes in the brain. Developmental exposure to BPA alters the DNA methylation promoter state in many genes of the mouse forebrain (Yaoi et al., 2008). Persistent DNA methylation changes are evident in one of the promoters of *Bdnf* in the hippocampus and cord blood in female and male mice subjected to prenatal exposure to BPA with hypemethylation apparent in females but hypomethylation observed in males (Kundakovic et al., 2014). DNA methyl transferase (DNMT) and methyl CPG binding proteins (MECP) guide global DNA methylation changes.

BPA may simultaneously effect DNA methylation and histone protein modifications. For instance, exposure to BPA suppresses rat brain cortical expression of the ion transporter (*Kcc2*) through both DNA methylation via MECP2 and histone protein (H3K9) binding to this gene (Yeo et al., 2013). Phthalates can also induce histone protein modifications in neuronal cells, as evidenced by phthalate-induced Sp3 suppression associated with deacetylation (via HDAC4) and ensuing polyubiquination in neuroblastoma cells (Guida et al., 2014).

The expression of *Dnmt1*, *Dntm3a*, *Dnmt3b*, and *Mecp2* is altered in various brain regions, including basolateral amygdala, cortex, and hypothalamus, in BPA-treated rodent models (Kundakovic et al., 2013; Warita et al., 2013; Zhou et al., 2013). The histone protein modification enzyme (HDAC2) is up-regulated in the hippocampus of adult males exposed to BPA (Zhang et al., 2014a). The histone methyltransferase enzyme (EZH2) and histone H3 trimethylation is upregulated in the mammary gland of mice exposed to BPA *in utero* and MCF-7 cells treated with this chemical (Doherty et al., 2010).

While no study has determined whether BPA exposure affects non-coding (nc) RNAs (including microRNAs, miRNAs) in the brain, there is evidence that this chemical can affect expression of these biomolecules in other cells, including MCF7 (Tilghman et al., 2012), ovarian (Veiga-Lopez et al., 2013), and placental cells (Avissar-Whiting et al., 2010). No study to date has assessed whether phthalate exposure affects ncRNAs in the brain or other regions.

## Conclusions

Strong evidence exists that parental and social behaviors in a wide variety of species, including by translation humans, are vulnerable to perinatal exposure to EDCs, including BPA and phthalates. These effects are likely multifactorial in origin, but the net result is presumably that these chemicals disrupt normal organizational and activational programming of the brain. EDC-induced disruptions on neural programming may occur as a result of alterations in fetal or adult steroid hormone production, steroid receptor expression in the brain regions governing these traits, neuropeptide/protein hormones and their cognate receptors, and/or through epimutations. It remains to be determined though whether BPA, generally considered a “*weak estrogen*,” results in similar mechanistic disruptions as phthalates with their predominantly anti-androgenic effects. Both chemicals can potentially disrupt all of the above pathways. Further, it is now apparent that both chemicals may lead to pleiotropic disturbances through steroidogenic and non-steroidogenic pathways (Leon-Olea et al., 2014).

EDC-induced parental and social behavioral deficits could affect the general livelihood, social well-being, ability to attract mate(s), reproduction, and likelihood of successfully rearing young. Thus, early exposure of animal and humans to these widely prevalent chemicals may lead to insidious behavioral effects. Moreover, there is ample evidence in animal models that EDCs (and humans in the case of DES) can induce transgenerational effects (Newbold et al., 2000, 2006; Klip et al., 2002; Titus-Ernstoff et al., 2006; Wolstenholme et al., 2012, 2013; Doyle et al., 2013; Manikkam et al., 2013; Schneider et al., 2013; Zhang et al., 2014b). Therefore, these chemicals might already be exhuming a toll on the social lives of unborn generations. While a call to action to legislate further these chemicals seems a reasonable course of action based on the existing data and precautionary principle, identifying the underpinning mechanisms leading to these behavioral disturbances might provide the incentive for policymakers to act. Long-term studies examining for linkages in parental deficiencies and transgenerational effects in human populations exposed to these EDCs may regrettably be needed before the production of such chemicals is minimized or outright banned.

### Conflict of interest statement

The author declares that the research was conducted in the absence of any commercial or financial relationships that could be construed as a potential conflict of interest.
